# Role of personal resources from the perspective of experiencing tinnitus annoyance in adults

**DOI:** 10.1007/s00405-020-05843-w

**Published:** 2020-02-26

**Authors:** Małgorzata Fludra, Joanna Kobosko, Elżbieta Gos, Karina Karendys-Łuszcz, Henryk Skarżyński

**Affiliations:** 1 Rehabilitation Clinic, World Hearing Center, Institute of Physiology and Pathology of Hearing, Warsaw, Poland; 2 Teleaudiology and Screening Department, World Hearing Center Institute of Physiology and Pathology of Hearing, Warsaw, Poland; 3 Tinnitus Clinic, World Hearing Center, Institute of Physiology and Pathology of Hearing, Warsaw, Poland; 4 Oto-Rhino-Laryngology Surgery Clinic, World Hearing Center, Institute of Physiology and Pathology of Hearing, Warsaw, Poland

**Keywords:** Positive orientation, Basic hope, Tinnitus

## Abstract

**Purpose:**

Occurrence of tinnitus can be, for a person who experiences it, a disorder affecting the overall equilibrium of the organism. To cope with it a variety of personal resources, such as positive orientation and basic hope, are mobilized. The aim of this study was to determine whether these resources are associated with the evaluation of the impact of tinnitus on the functioning of the study participants.

**Methods:**

Study involved 176 tinnitus sufferers, including 123 women and 53 men. The results were compiled using: Positivity Scale (P*-*scale) for measuring positive orientation, Basic Hope Inventory (BHI-R) for measuring basic hope, the questionnaires: Tinnitus Handicap Inventory (THI) and Tinnitus Functional Index (TFI) evaluating the impact of tinnitus on the daily functioning of the subjects, as well as a survey designed for the study, comprising questions about sociodemographic data and tinnitus history.

**Results:**

Results of regression analysis indicated that positive orientation is the most important for the perceived tinnitus annoyance. However, no significant influence of basic hope on tinnitus annoyance was found. Among other variables taken into account in regression analysis, age and presence of additional diseases proved to be important predictors of tinnitus annoyance.

**Conclusions:**

Personality determinants (positive orientation) are related to the perception of tinnitus annoyance. When working with a tinnitus patient, it is justified to pay attention to the existence of the positive orientation and to work on its development. Research should also be continued to search for other personal resources that affect the perceived tinnitus annoyance.

## Introduction

Holistic-functional model defines health as the process of continuous balancing of human needs and environmental requirements. Disease is, therefore, the exceeding of the limits of an acceptable state of imbalance. The model also assumes that humans have resources/potentials enabling them to adapt to the changing conditions of both the internal and external environment [1–3]. Therefore, it shows how important is the role played by the resources possessed by humans in their functioning. These resources, following Hobfoll the creator of the COR Conservation of Resources Theory, can be described as “objects, conditions, personality traits and energy deposits, which are either valued in themselves as necessary for survival (directly or indirectly) or are used to obtain those resources enabling survival”.[4].

Occurrence of tinnitus, as shown by research, may affect the quality of life of a person experiencing it, their emotional state, concentration and attention, as well as rest and sleep [5–9]. Therefore, it may lead to a potential loss disrupting the existing equilibrium of the organism. To cope with it, the person experiencing such a loss uses the resources available to them. These resources influencing coping with chronic diseases and ailments include, among others, positive orientation and basic hope.

Positive orientation is a relatively new psychological construct designed by Gian Vittorio Caprara. It integrates three components: self-esteem, life satisfaction and optimism. Positive orientation is a personality trait that manifests itself in a tendency to perceive life, the future and oneself with a positive attitude. Positive orientation is treated as the opposite of the so-called Beck's depressive triad [10–12]. According to Caprara's research, it correlates positively with the perceived state of health, which means that a higher level of this variable has an influence on a more positive assessment of health [13]. In other research studies, the same author proves that cancer patients with a higher level of positive orientation report fewer physical and mental symptoms than patients with its lower level [14]. As shown in Byra’s research, positive orientation has an impact on the level of posttraumatic growth and positive adaptation among people with spinal cord injury [15].

Basic hope is a personal resource understood, according to Erikson's theory, as a fundamental belief of an individual in an orderly and meaningful world and its positivity towards people. Basic hope is shaped in childhood on the basis of the child’s experience with primary caregivers, mainly the mother [16]. According to Trzebiński and Zięba, basic hope is a relatively stable personality structure of a cognitive-emotional nature, which allows an individual to respond constructively in situations of novelty or loss of the existing order. It determines the way of dealing with existential dilemmas. A high level of basic hope means a strong belief in the order and positivity of the world, which makes it possible to accept a loss, such as loss of health. It allows to stop spending energy and strength in the situation of loss, thus enabling both cognitive and emotional construction of the present and future [17]. The research has shown that the high level of basic hope in people with permanent disability as a result of traffic accidents increased the positive reframing of this critical event [18]. Among women with breast cancer and those after mastectomy, the high level of this variable was associated with better emotional coping with the situation of trauma, facilitating the patients' activation of the remaining resources, thus enabling them to adapt to the situation and build a new order in life [19]. Basic hope is also an important resource in new life situations, i.e. in situations of building a new order. These include for example marriage and the birth of the first child. Terelak and Demkiewicz's research proves that strong basic hope in pregnant women correlates with the use of adaptive stress coping strategies [20].

Positive orientation and basic hope play an adaptive role and motivate an individual to act. They seem to be convergent in meaning, but as Łaguna's research shows, although there is a positive correlation between the P Scale (measuring positive orientation) and the BHI-12 Basic Hope Inventory (*r* = 0.51, *p* = 0.001), they are separate dimensions [21].

The aim of this study was to determine whether positive orientation and basic hope are associated with the evaluation of the impact of tinnitus on the functioning of the subjects. The authors of this article were unable to find any research studies describing the impact of the personal resources selected for this study on tinnitus annoyance in the available literature.

Therefore, the following research questions were posed:

- Is positive orientation connected with tinnitu annoyance?
- Does basic hope have an impact on the perceived tinnitus annoyance?

## Material

Study involved 176 tinnitus sufferers, including 123 women and 53 men aged 31–80 (*M* = 59.10; SD = 9.56). The majority of subjects had higher than secondary education (58%), were professionally active (58%), lived in big cities (67%) and were either married or in informal relationships (72%).

Duration of tinnitus ranged from half a year to 30 years (*M* = 8.34, SD = 6.12). Normal hearing in both ears, based on tonal audiometry (arithmetic mean of hearing threshold values for 500, 1000, 2000, 4000 Hz), was found in 80 (45.5%) subjects. The remaining 96 (54.5%) had unilateral or bilateral hearing loss of at least mild degree.

## Method

Positive orientation was measured using the Positivity Scale (P Scale) developed by Caprara and colleagues, in the Polish adaptation by Łaguna, Oleś and Filipiak [21]. The tool consists of eight statements to which the study participant responds on a five-point Likert scale (from 1–definitely disgree to 5–definitely agree). The overall score that can be obtained ranges from 8 to 40 points. The higher the score obtained on the questionnaire, the higher the level of positive orientation.

Level of basic hope was assessed using the BHI-R by Trzebiński and Zięba [22]. The tool is composed of 20 statements, including 16 diagnostic ones. The study participant chooses one of the five possible answers on the scale: from 1–definitely disagree to 5–definitely agree. The overall score is the sum of the points obtained from the answers to diagnostic items and ranges from 16 to 80 points. A higher score means a higher level of the variable.

Study also used the Tinnitus Handicap Inventory (THI) by Newman in the Polish adaptation by Skarżyński, whose aim is to assess the impact of tinnitus on the daily functioning of the subjects [23, 24]. The questionnaire consists of 25 items. In each of them the study participant gives one of the three answers: yes (four points), sometimes (two points), no (zero points). THI is divided into three subscales: Functional, Emotional and Catastrophic. The Functional subscale is designed to assess the impact of tinnitus on social, cognitive and physical functioning. The Emotional subscale shows emotional reactions to tinnitus. The Catastrophic one describes catastrophic reactions of the subjects to tinnitus, e.g.: do you feel that you can no longer stand your tinnitus? The sum of the points obtained from all the subscales determines one of the five degrees of tinnitus annoyance: level 1 (0–22 points) means little or no tinnitus impact on the daily functioning of the patient, level 2 (24–48 points) low to moderate impact, level 3 (50–72 points) strong tinnitus, level 4 (74–100 points) very strong tinnitus.

Tinnitus Functional Index (TFI) by Meikle and others [25] was also used to assess the impact of tinnitus on everyday functioning. The questionnaire is divided into eight subscales: intrusiveness, sense of control, cognitive functioning, sleep, hearing, rest, quality of life and emotions. The subject fills in 25 positions, of which three positions fall into each of the eight categories (apart from the quality of life). The answer is given on a scale from 0 to 10. Extreme values are described in words in such a way as to be a reference point for the subject to determine their current feelings as accurately as possible. The scores are then calculated according to the instructions. The maximum score for the whole questionnaire is 100 points. Also, 100 points can be obtained on each scale. The subjects also completed a survey comprising questions about sociodemographic data and information related to the history of the ailment.

To investigate the relationship between basic hope, positive orientation and tinnitus annoyance, correlation and regression analyses were performed. The level of significance was *α* = 0.05. For statistical analyses, IBM SPSS Statistics was used (v. 24).

## Results

### Level of positive orientation and basic hope

In the whole study group of people with tinnitus the level of positive orientation ranged from 16 to 40 points, averaging 29.09 (SD = 4.67), i.e., the fifth sten according to Polish standards for the P Scale. Basic hope assumed values ranging from 36 to 76 points, averaging 58.64 (SD = 7.63), i.e., the sixth sten according to temporary sten norms for the general population.

Positive orientation and basic hope were significantly correlated with each other: *r* = 0.43; *p* < 0.001. The direction of the correlation was positive, i.e. higher values of positive orientation were accompanied by higher values of basic hope.

Impact of sociodemographic variables: gender, age, education, professional activity, partner/marital status, place of residence, and health-related variables: tinnitus duration, hearing loss (division due to lack/occurance of hearing loss), presence or absence of other diseases, on the level of positive orientation and basic hope was studied. Additionally, correlation coefficients between positive orientation and age were determined: *r* = 0.20; *p* < 0.01 and between positive orientation and tinnitus duration: *r* = 0.08; *p* > 0.05 as well as between basic hope and age: *r* = 0.05; *p* > 0.05 and between basic hope and tinnitus duration *r* = 0.08; *p* > 0.05.

Analysis shows that only age was significantly correlated with the level of positive orientation. The correlation had a positive direction, i.e. the level of positive orientation increased with age. In the case of basic hope, none of the variables was significantly correlated with its level.

### Level of tinnitus annoyance

Tinnitus annoyance measured with the THI questionnaire ranged from 2 to 98 points, averaging 44.78 (SD = 21.13) so in accordance to Polish norm for the population of tinnitus sufferers, tinnitus had a low to moderate level in the studied group.

Tinnitus annoyance measured with the TFI questionnaire ranged from 3 to 90 points, averaging 42.52 (SD = 19.82). This means that, on average, tinnitus had a moderate impact (3rd degree of annoyance) on the lives of the subjects.

### Relationship between positive orientation, basic hope and tinnitus annoyance

Relationship between positive orientation, basic hope and tinnitus annoyance was investigated by means of correlation analysis. The *r*-Pearson correlation coefficients between the variables are presented in Tables 1 and 2.

**Table 1:** Correlations between positive orientation, basic hope and tinnitus annoyance (THI)

	*F*	*E*	*C*	THI
Positive orientation	− 0.30**	− 0.30**	− 0.14	− 0.30**
Basic hope	− 0.11	− 0.19*	0.04	− 0.14

*THI* overall score THI, *F* functional scale score, *E* emotional scale score, *C* catastrophic scale score

^**^p < 0.01, *p < 0.05

**Table 2:** Correlations between positive orientation, basic hope and tinnitus annoyance (TFI)

	Int	*C*	Cogn	*S*	*H*	*R*	QoL	*E*	TFI
Positive orientation	− 0.09	− 0.24**	− 0.33**	− 0.11	− 0.12	− 0.19*	− 0.21**	− 0.28**	− 0.24**
Basic hope	− 0.08	− 0.10	− 0.11	− 0.07	− 0.05	− 0.06	− 0.11	− 0.22**	− 0.12

*TFI* General score; Scale score, *Int* intrusiveness, *C* control, *Cogn* cognitive functioning, *S* sleep, *H* hearing, *R* relaxation, *QoL* quality of life, *E* emotions

^**^p < 0.01, *p < 0.05

Positive orientation was significantly and negatively related to tinnitus annoyance measured with THI questionnaire. The higher the level of positive orientation, the lower the level of tinnitus annoyance in the general, functional and emotional dimensions. There was also a significant, negative, though very weak correlation between basic hope and tinnitus annoyance in the emotional dimension.

Positive orientation was significantly and negatively related to tinnitus annoyance as measured with the TFI questionnaire. The higher the level of positive orientation, the lower the level of tinnitus annoyance in the general dimension and in the dimensions of control, cognitive functioning, relaxation, quality of life and emotions. There was also a significant negative correlation found between basic hope and tinnitus annoyance, but only in the emotional dimension.

Using regression analysis, the combined effect of positive orientation and basic hope on tinnitus annoyance was investigated. The potential influence of independent sociodemographic and biomedical variables (age, presence of additional illnesses, duration of tinnitus, education), as well as positive orientation and basic hope were also taken into account. In the first step, selected independent sociodemographic and biomedical variables were included in the model, in the second step, positive orientation was included in the model, and in the third step–basic hope was included in the model. Each time the significance of the model and the change of the explained variability were examined. Table 3 presents the results of regression analysis for tinnitus annoyance measured with THI and TFI questionnaires.

**Table 3:** Regression coefficients for tinnitus annoyance (THI and TFI) depending on positive orientation, basic hope and selected sociodemographic and biomedical variables

	THI			TFI		
	*β*	*t*	*p*	*β*	*t*	*p*
Age	− 0.16	− 1.99	0.048	− 0.04	− 0.56	0.576
Presence of illness	− 0.15	− 1.90	0.059	− 0.11	− 1.42	0.156
Duration of tinnitus	0.14	1.80	0.074	0.14	1.81	0.072
Education	− 0.12	− 1.60	0.111	− 0.16	− 2.14	0.034
Positive orientation	− 0.27	− 3.27	0.001	− 0.23	− 2.81	0.005
Basic hope	− 0.01	− 0.13	0.897	− 0.02	− 0.19	0.853

*β* standardized coefficient of regression, *t* value of test statistic, *p* statistical significance

In the case of tinnitus annoyance measured with the THI questionnaire, the first regression model, which included only selected independent sociodemographic and biomedical variables, was statistically significant: *F*(4,171) = 4.10; *p* < 0.01; *R*^2^ = 0.066, adjusted *R*^2^ = 0.058. The second model with the previously included variables and positive orientation *F*(5,170) = 6.24; *p* < 0.001; *R*^2^ = 0.155, adjusted *R*^2^ = 0.130 was also significant. After the inclusion of positive orientation into the model, the explained variability increased: Δ*R*^2^ = 0.068. The third model included all the previous variables and basic hope *F*(6,169) = 5.18; *p* < 0.001; *R*^2^ = 0.155, adjusted *R*^2^ = 0.125. In this case, there was no increase in the explained variability (Δ*R*^2^ = 0.000). Table 3 presents the regression coefficients obtained for the third model. They show that positive orientation is of key importance for tinnitus annoyance. Based on the high level of this variable, reduced tinnitus annoyance can be predicted. Among sociodemographic and biomedical variables, age turned out to be a significant predictor with age the declared tinnitus annoyance decreased. Also the presence of illnesses was of some importance. Healthy individuals tended to feel less tinnitus annoyance. However, no significant effect of basic hope on tinnitus annoyance measured with the THI questionnaire was observed.

In the case of tinnitus annoyance measured with the TFI questionnaire, the first regression model (only with selected sociodemographic and biomedical variables) was statistically significant: *F*(4,171) = 3.56; *p* < 0.01; *R*^2^ = 0.077, adjusted *R*^2^ = 0.055. The second model containing the previously included variables and positive orientation was also significant: *F*(5,170) = 5.06; *p* < 0.001; *R*^2^ = 0.129, adjusted R^2^ = 0.104, and an increase in the explained variability was recorded Δ*R*^2^ = 0.053. The third model containing all previous variables and basic hope was statistically significant: *F*(6,169) = 4.20; *p* < 0,01; *R*^2^ = 0.130, adjusted R^2^ = 0.099, but there was no increase in the explained variability (Δ*R*^2^ = 0.000) in it.

Analysis of the values of regression coefficients and their significance leads to the conclusion that positive orientation is of the greatest importance for the perceived tinnitus annoyance. Based on the high level of this variable, a reduced tinnitus annoyance can be predicted. Among the selected sociodemographic and biomedical variables, education was also an important predictor (people with higher education declare less tinnitus annoyance). However, no significant impact of basic hope on tinnitus annoyance measured with the TFI questionnaire was found, similarly as in the case of THI questionnaire.

## Discussion

Majority of studies on tinnitus so far have focused on risk factors increasing the likelihood of difficulties in adapting to the condition. In recent years, due to both positive and health psychology, there has been an increasing focus on finding factors that promote health and better coping with experienced health problems. The aim of the study as part of this trend was to assess the importance of selected personal potentials, i.e. positive orientation and basic hope in the subjectively perceived impact of tinnitus on the daily functioning of people suffering from this ailment.

Due to the fact that the investigation of basic hope was conducted using a new version of the BHI-R, its correlation with the P-Scale used to evaluate positive orientation was checked. As in Łaguna’s study on the P and BHI-12 scales, the correlation proved to be positive and statistically significant but excluded the possibility that these tools examine the same psychological construct [21].

Obtained results confirm the significant influence of positive orientation on the evaluation of tinnitus annoyance using both THI and TFI questionnaires. On their basis, less tinnitus annoyance can be expected in people with a higher level of positive orientation. This result shows that positive orientation is a protective factor in the event of tinnitus, i.e. a health-related stressful situation. It allows to see the future in a more positive way, anticipate the possibility of coping with the ailment and appreciate the current situation despite the occurrence of the ailment, thus influencing the anticipation of the consequences of tinnitus on the daily functioning of people experiencing it. The obtained results are consistent with Caprara’s research, which shows how important is a high level of positive orientation in alleviating the assessment of one's own health in old age. Elderly people with high levels of this variable reported fewer health problems [26]. One of the first studies by Caprara also showed that a higher level of this variable is associated with a perceived better quality of health and interpersonal relations, as well as with a hedonistic equilibrium [13]. Further studies of the Caprara and Alessandri’s team have proved that positive orientation paves the way for positive affectivity [27, 28], which, as shown by Frederickson’s studies [29], is a determinant of a good quality of life.

Basic hope was the second personal resource studied. In the case of this resource, only weak positive correlations with the emotional scale in the THI questionnaire and with the dimension of emotions in the TFI questionnaire were obtained. In regression analysis, basic hope did not reduce tinnitus annoyance. In the available literature there are few studies on the impact of basic hope on adaptation to disease. The available ones concern diseases related to trauma associated with a threat to life or experienced disability [18, 19]. According to these studies, basic hope is an essential resource of an individual at the time of occurrence of a loss compromising the basic system of values. It seems that the concept of basic hope has a broader scope than positive orientation since it refers to the universe and the general basic concept of the world and the laws that govern it [30]. The personal resource in the form of basic hope is activated at the moment of the disintegration of the existing order, triggering the knowledge and beliefs contained in basic hope concerning the foundations of reality. Thus, it seems that the occurrence of tinnitus, despite its chronic character and lack of possibility, in most cases, of completely eliminating it, is not such a breakthrough event in life for those who experience it, which would require activation of basic hope.

Discussed study indicates the importance of personality determinants of the perception of tinnitus annoyance. However, it should be remembered that the age of tinnitus sufferers and their general health status are related to the perception of tinnitus annoyance. The study participants declaring experiencing additional chronic diseases tend to perceive tinnitus as more troublesome. Such dependence may be explained, according to Hobfol's theory of conservation of resources, by the burden associated with coping with another ailment (use of resources) and, consequently, by the lower availability of resources that can be used in the process of coping with tinnitus. Also younger subjects feel greater impact of this ailment on everyday life than the older ones. This result is surprising and contradicts other reports in the literature [31–33].

In the context of psychological and psychotherapeutic work, the question arises whether positive orientation can be developed. The research conducted by Caprara [12] indicates a fairly high heritability rate of this trait. Fegnani [34] notes that the average level of positive orientation increases over the course of life and estimates its heritability at 58%, which means that environmental factors are also of significant importance. In her article, Łaguna [35] presents a theoretical mechanism for the development of positive orientation. She presents the B-A-E model (belief-affect-engagement), according to which positive orientation influences momentary positive beliefs, which in turn stimulate positive affect. Such positive affective experiences may then build more stable aspects of affective states, and these may consequently contribute to building lasting positive affectivity at the level of a trait. This mechanism is a complement to the reverse mechanism in which stable traits affect momentary experiences. The Łaguna model explains changeability, moderate plasticity of the positive orientation over time, confirmed also in this study, and at the same time shows the direction of the therapeutic work. Other authors also point to self-efficacy as an intermediate variable affecting positive orientation [36].

In the conducted study positive orientation, but not basic hope, proved to play a significant role in how tinnitus annoyance was perceived. This result indicates also the personality determinants of the perception of tinnitus annoyance. It should be remembered, however, that the age and education of tinnitus sufferers and their general health status are related to how tinnitus annoyance is perceived. The P-scale is an easy to use tool as it takes about 1 min to complete, so it seems to be the questionnaire useful in everyday clinical practice as a screening tool assessing the probability of easier adaptation to tinnitus. It is also justified to include methods strenghtening self-efficacy in psychological therapy of patients suffering from significant tinnitus annoyance and having difficulties in adapting to this ailment.

## Conclusions

- Positive orientation remains in relation to the perception of tinnitus annoyance. When working with a tinnitus patient, it is justified to pay attention to the presence of this resource and to work on its development.
- No significant impact of basic hope on tinnitus annoyance was found. Further research should be continued to search for other personal resources that may affect the perceived tinnitus annoyance.
